# Italian regional data and oropharyngeal adverse events to drugs and vaccines: insights from a 3-year spontaneous reporting (2022–2024)

**DOI:** 10.1007/s43440-026-00873-3

**Published:** 2026-06-15

**Authors:** Liberata Sportiello, Mario Gaio, Alessia Zinzi, Gaetano La Mantia, Vera Panzarella, Giuseppe Colella, Paola Martina Marra, Laura Sottosanti, Simona Potenza, Giacomo Oteri, Martina Coppini, Giuseppe Seminara, Rodolfo Mauceri, Ilaria Morreale, Vittorio Fusco, Giuseppina Campisi, Annalisa Capuano

**Affiliations:** 1 Campania Regional Centre for Pharmacovigilance and Pharmacoepidemiology, University of Campania “Luigi Vanvitelli”, Naples, Italy; 2 Department of Experimental Medicine, University of Campania “Luigi Vanvitelli”, Naples, Italy; 3 Department of Life Science, Health, and Health Professions, Link Campus University, Roma, Italy; 4 Unit of Oral Medicine and Dentistry for Fragile Patients, Department of Rehabilitation, Fragility, and Continuity of Care, University Hospital Palermo, Palermo, Italy; 5 Department of Precision Medicine in Medical, Surgical and Critical Care (Me.Pre.C.C.), University of Palermo, Palermo, Italy; 6 Department of Biomedical and Dental Sciences and Morphofunctional Imaging, University of Messina, Messina, Italy; 7 Oral and Maxillofacial Surgery Unit, Multidisciplinary Department of Medical - Surgical and Dental Specialties, University of Campania “Luigi Vanvitelli”, Naples, Italy; 8 Complex Operative Unit of Stomatological surgery in Developmental age, University of Campania “Luigi Vanvitelli”, Naples, Italy; 9 Italian Medicines Agency, Rome, Italy; 10 Department of Precision Medicine in Medical, Surgical and Critical Care, University of Palermo, Palermo, Italy; 11 Internal Medicine, Pharmacovigilance and Clinical Pharmacology Unit, Department of Promoting Health, Maternal- Infant, Excellence and Internal and Specialized Medicine (PROMISE) G. D’Alessandro, Sicilian Regional Pharmacovigilance Centre, University of Palermo, Palermo, Italy; 12 Oncology Unit, Department of Medicine and Translational Medicine Unit, DAIRI - Department of Integration, Research and Innovation, “SS Antonio e Biagio e C. Arrigo” Hospital, Alessandria, Italy; 13 Department of Biomedicine, Neuroscience and Advanced Diagnostic, University of Palermo, Palermo, Italy

**Keywords:** Oropharyngeal adverse events, Medication- related osteonecrosis of the jaw, Dentists, Pharmacovigilance, Spontaneous reporting, Database

## Abstract

**Background:**

Oropharyngeal adverse events (O-AEs) represent a potential safety concern associated with several drugs and/or vaccines. Although often underestimated, these events may provide valuable insights into a patient’s overall clinical condition, appearing initially mild but later worsening. Therefore, this study aimed to analyze O-AEs related to drugs and/or vaccines using structured safety data.

**Methods:**

Safety reports from three Italian regions were retrieved from the national pharmacovigilance database (*rete nazionale di farmacovigilanza*, RNF) and analyzed for the period 2022–2024. All reports were structured and analyzed according to the International Council of Harmonisation (ICH) E2B (R3) format. Additionally, reporting odds ratios (RORs) were calculated to compare the likelihood of O-AEs being reported by different categories of reporters (e.g., physicians, other healthcare professionals, or patients).

**Results:**

Over three years, 47,664 reports were collected, of which 1,740 (3.6%) contained at least one suspected O-AE. Most patients were female (65.7%) with a median age of 55 years (IQR 38–66). The majority of reports described non-serious events (66.7%), and outcomes were favorable in most cases (75.9%). The safety reports related to drugs (90.5%) were largely more than those related to vaccines (9.5%). A total of 129 cases of medication-related osteonecrosis of the jaw (MRONJ) were identified, most of which were serious and had unfavorable outcomes. Disproportionality analysis revealed that physicians were less likely to report O-AEs than patients (ROR=0.72; 0.62-0.84; P<<0.05) and more likely than nurses (ROR=1.34; 0.98-1.87; P<0.05). Compared with 2019–2021, the main difference was the lower proportion of vaccine-related reports, which declined from 47.8% to 9.5% in 2022–2024.

**Conclusions:**

Only a small proportion of safety reports involved O-AEs. These findings highlight the importance of enhancing awareness among physicians (particularly dentists) regarding O-AEs, and of fostering a collaborative pharmacovigilance culture across healthcare providers, thereby improving patient safety through more timely and reliable reporting.

**Supplementary Information:**

The online version contains supplementary material available at 10.1007/s43440-026-00873-3.

## Introduction

Oropharyngeal adverse events (O-AEs) can pose a safety issue associated with the use of various medications, including anti-infectives for systemic use, antineoplastic and immunomodulating agents [1–3]. Such manifestations include a wide range of clinical conditions, such as throat tightness, oral paresthesia, oropharyngeal pain, dysphagia, and angioedema, which can occur in isolation or in association with other systemic effects [4]. Among the most severe adverse reactions associated with the use of systemic action drugs, medication-related osteonecrosis of the jaw (MRONJ) stands out, particularly that of the jaws, a rare but well-documented complication that can occur following treatment with antiresorptive agents such as bisphosphonates, biologic agents such as denosumab, or, less commonly, with angiogenesis inhibitors. The O-AEs, although often overlooked, can provide relevant information about the overall clinical status of the patient [5, 6]. Recognizing them early is essential, as they can significantly influence the course of the disease [4]. The prognosis of the O-AEs, in fact, varies based on the timeliness of the diagnosis, the stage of the disease, and the response to therapies. In this context, pharmacovigilance, through the collection and analysis of spontaneous reporting of suspected adverse drug reactions (ADRs), plays an essential role in the early identification of such events [7]. Despite the increasing attention to monitoring adverse events (AEs), those affecting the oral cavity continue to be underreported and often overlooked, reflecting a persistent gap in awareness among healthcare professionals (HCPs) [8]. In fact, the underreporting of the O-AEs is often attributed to a lack of awareness and the absence of adequate training among HCPs. Moreover, the data from the literature highlight that HCPs are more inclined to primarily report serious or unexpected AEs, overlooking those that are less severe but still relevant for monitoring drug safety [9]. Although still a largely unexplored area, especially by oral HCPs, our previous analysis examined the contribution of HCP sand a total of 2773 reports of O-AEs collected in the Italian pharmacovigilance database in a 3-year study period (2019–2021) [4]. Considering the study period and the potential “masking effect” on some AEs caused by the substantial increase in reports related solely to coronavirus disease-19 (COVID-19) vaccines across various spontaneous reporting systems, we conducted a descriptive analysis of ICSRs (individual case safety reports) submitted in three Italian region (Piedmont, Sicily, and Campania) over three years 2022–2024, using data from the Italian national pharmacovigilance system (*rete nazionale di farmacovigilanza*, RNF). Furthermore, we focused the analysis on the potential cases of MRONJ. Finally, we also aimed to evaluate if HCPs have a lower/higher probability of reporting O-AEs in a direct comparison with the other sources (i.e., patients, pharmacists, nurses, and other HCPs).

## Methods

### Data source

The RNF is the official system for collecting ICSRs of AEs associated with drugs or vaccines. Established in 2001 and managed by the Italian medicines agency (AIFA), the RNF serves as a key tool for the post-marketing surveillance of medicinal products. The RNF is fully integrated with the European Medicines Agency’s EudraVigilance (EV) database and adheres to the standardized International Council of Harmonisation (ICH) E2B (R3) format for reporting, ensuring compatibility with international pharmacovigilance reporting requirements [10]. The ICH E2B (R3) defines the data elements, message specifications, and structure required for the standardized exchange of ICSRs between pharmaceutical companies and regulatory authorities. This format was introduced in Europe in 2017 and officially adopted in 2022.

Access to safety data from the RNF is restricted and granted only to authorized stakeholders, including Ministry of Health, the *Istituto Superiore di Sanità*, regional regulatory authorities, regional pharmacovigilance centers, public sector qualified persons for pharmacovigilance (QPPVs), and marketing authorization holders. A dedicated section of the AIFA website (https://servizionline.aifa.gov.it/) provides these entities with secure access to selected information from the database.

Each ICSR includes a range of key information such as its origin (whether transmitted via EV or not), the date of data entry, patient demographics (e.g., sex and age), the qualification of the primary source, a list of reported AEs, and details on suspected, interacting, or concomitant drugs. Outcomes are categorized as recovered/resolved, recovering/resolving, recovered with sequelae, not recovered/not resolved, or fatal. Cases are also assessed for seriousness, classified either as non-serious or serious. Serious cases fall under specific criteria: death, life-threatening conditions, congenital anomalies, disabling events, or events leading to or prolonging hospitalization. Additionally, AEs that do not meet these criteria but are considered significant by the reporter or are listed in the EMA’s important medical events (IME) list are categorized as “other medically important condition”. The IME list is designed to support consistent classification of serious AEs across the European Union (EU) [11].

To ensure standardized reporting, the RNF employs the Medical Dictionary for Regulatory Activities (MedDRA) terminology for coding all signs, symptoms, and diagnoses associated with AEs. MedDRA is structured as a hierarchical classification system, beginning with broad categories known as system organ classes (SOCs) and progressing through increasingly specific levels. At the mid-level are preferred terms (PTs), which represent distinct medical concepts, and these are further broken down into the most detailed level, the low-level terms (LLTs) [12]. All data elements comply with ICH E2B(R3) requirements for electronic transmission.

To support regulatory monitoring, MedDRA includes standardized MedDRA queries (SMQs), which are pre-defined collections of related terms (i.e., PTs) grouped around specific medical conditions or areas of safety concern, such as oropharyngeal disorders. Some SMQs consist of flat lists of PTs, while others are organized hierarchically and contain nested, more focused SMQs.

### Data queries

**Fig. 1:**
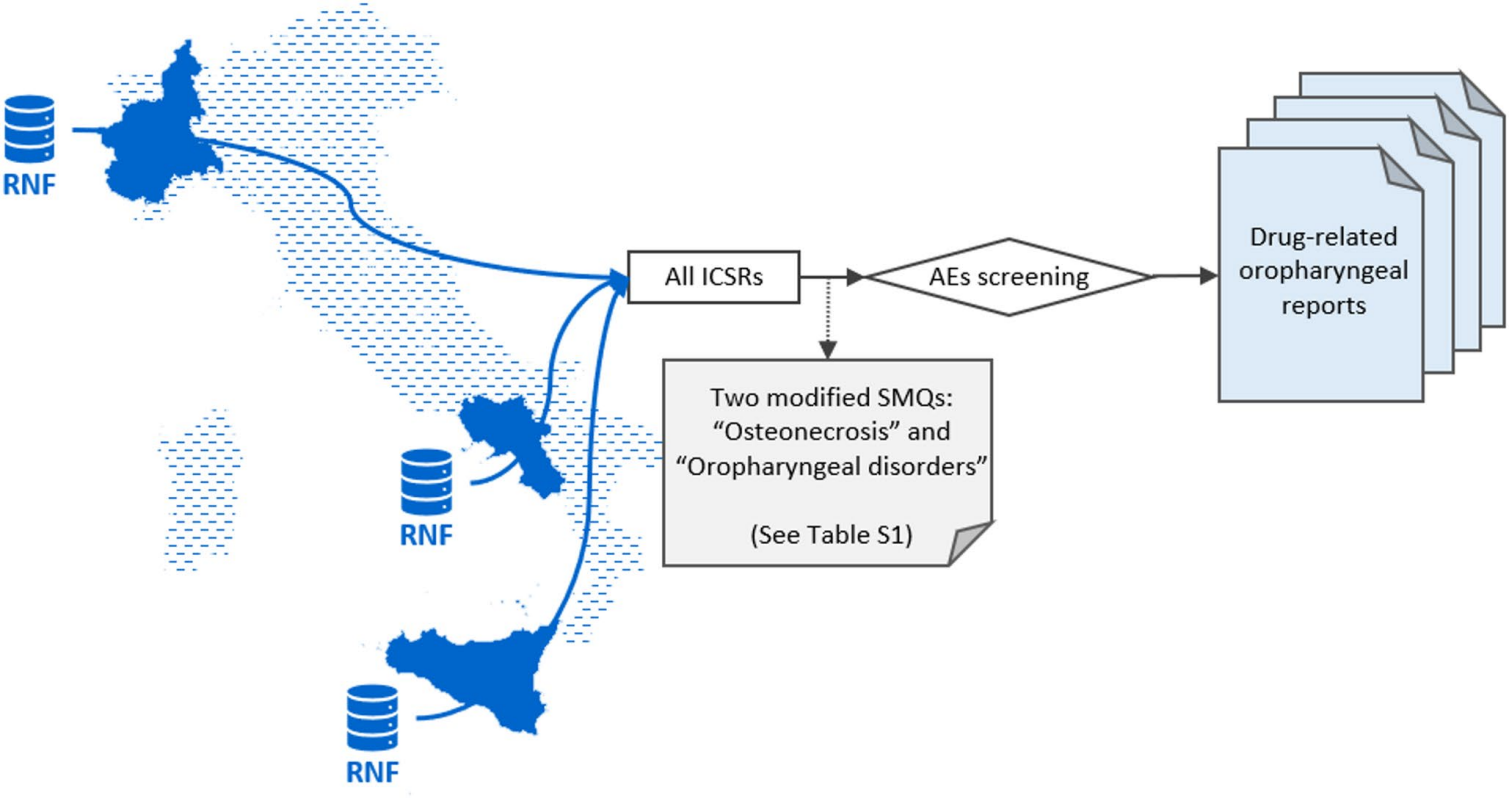
Flowchart of data collection of drug-related oropharyngeal individual case safety reports (ICSRs) collected in the Italian National Pharmacovigilance Network (*Rete Nazionale di Farmacovigilanza*, RNF) by three Italian regions (i.e., Piedmont, Campania, and Sicily) in the period 2022–2024. *AE* adverse event, *ICSR* individual case safety report, *RNF rete nazionale di farmacovigilanza*, *SMQ* standardized MedDRA query

Three distinct Italian regions – Piedmont, Campania, and Sicily – independently extracted all ICSRs submitted within their respective territories between January 1, 2022, and December 31, 2024. This process was carried out with the support of each regional pharmacovigilance center. The regional datasets were then merged to create a single dataset for analysis. From this combined dataset, only ICSRs containing at least one AE involving the oropharyngeal area were selected for each region (Fig. 1). This selection was based on two adapted SMQs from MedDRA version 26.0: “osteonecrosis” and “oropharyngeal disorders”. The latter also included five specific sub-SMQs: “oropharyngeal neoplasms”, “oropharyngeal infections”, “oropharyngeal allergic conditions”, “gingival disorders”, and “oropharyngeal conditions (excluding neoplasms, infections, and allergies)” (MedDRA, version 26.0) [12].

The refinement of the full list of PTs derived from the selected SMQs was carried out independently by two experts with specialized knowledge in dentistry. The modified list of PTs is presented in Table S1.

### Data analysis

We performed a descriptive analysis only on O-AEs reported in the selected ICSRs, excluding all the other AEs not reported in our modified list of PTs derived from the prespecified SMQs.

All included ICSRs were analyzed based on several variables: total number of reports, total number of relevant PTs, patient sex and age, seriousness of the event, outcome, reporter qualification, and the type of suspected or concomitant drugs involved. When specific information was missing, it was recorded as “not specified.” Additionally, all PTs were organized into six categories, aligned with the SMQs used in the analysis. The complete list of PTs within each group is provided in Table S1. For all available variables, we compared the data from the current analysis with the findings of a previous, analogous study conducted over the preceding three-year period (2019–2021) [4].

To identify which source was most involved in reporting O-AEs, we conducted a disproportionality analysis using the Reporting Odds Ratio (ROR) with 95% confidence intervals (CI). The analysis compared physicians, patients, pharmacists, nurses, and other HCPs. The ROR was determined as the ratio of the odds of cases reported by a specific source compared to cases reported by another source. Specifically, the ROR was calculated as (a/b)/(c/d), where ‘a’ represents the number of cases with O-AEs by the selected source, ‘b’ denotes the number of cases with PTs not included in our list of PTs, ‘c’ signifies the number of cases with O-AEs with the compared source, and ‘d’ indicates the number of cases with PTs not included in our list of PTs with the compared source.

The analysis compared physicians, patients (non-HCPs), pharmacists, nurses, and other HCPs. The ROR was used to assess disproportionality, calculated as the ratio of the odds of reporting O-AEs by one source compared to another. Specifically, ROR was computed using the formula (a/b)/(c/d), where: a = number of cases with O-AEs reported by the selected source, b = number of cases with other PTs (i.e., not classified as O-AEs) reported by the selected source, c = number of cases with O-AEs reported by the comparison source, d = number of cases with other PTs reported by the comparison source [13].

## Results

### Characteristics of the selected ICSRs

**Fig. 2:**
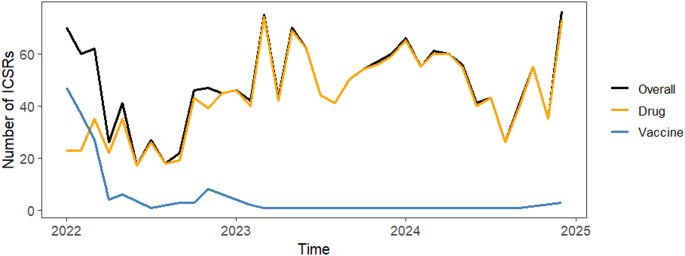
Trend of the total number of individual case safety reports (ICSRs) containing at least one oral adverse event (O-AE collected in the Italian National Pharmacovigilance Network (*rete nazionale di farmacovigilanza*, RNF) from 2022 to 2024 by three Italian regions (i.e., Piedmont, Campania, and Sicily), stratified by category of suspected drug (i.e., vaccine and drug)

In the 3-year study period (2022–2024), a total of 47,664 ICSRs were retrieved from the RNF: 24,802 for Campania, 14,324 for Sicily, and 8538 for Piedmont (Table 1). Among these ICSRs, 1740 fulfilled the selection criterion on the presence of at least one AE involving the oropharyngeal area. These selected cases represented 3.6% of the total ICSRs deriving from the three involved regions. In the considered period, the overall reporting contribution was about 43.0%, 39.2%, and 17.8% by Campania, Sicily, and Piedmont, respectively. The annual counts of the ICSRs reported during the period 2022–2024 are shown in Fig. 2. Most of the ICSRs recorded in the early months of 2022 referred to vaccines. However, this trend reversed in the following years: only 7 ICSRs in 2023 (1.1%) and 8 ICSRs in 2024 (1.3%).

**Table 1:** Characteristics of individual case safety reports (ICSRs) containing at least one oropharyngeal adverse event (O-AE), as collected in the Italian National Pharmacovigilance Network (*rete nazionale di farmacovigilanza*, RNF) between 2022 and 2024 by three Italian regions (i.e., Piedmont, Campania, and Sicily)

Characteristics	Level	Campania region	Sicily region	Piedmont region	Total (%)
ICSRs	Number	24,802	14,324	8538	47,664
ICSRs with O-AEs^a^	Number	748 (3.0)	683 (4.8)	309 (3.6)	**1740 (3.7)**
O-AEs^a^	Total number	793	764	325	**1882**
O-AEs^a^ per ICSR	Median (IQR)	2 (2–3)	3 (2–4)	2 (1–4)	**2 (1–4)**
Sex^b^	**Female**	468 (62.6)	459 (67.2)	216 (69.9)	**1143 (65.7)**
	Male	279 (37.3)	217 (31.8)	93 (30.1)	589 (33.9)
	Not specified	1 (0.1)	7 (1.0)	-	8 (0.4)
Age	Median (IQR)	54 (39–65)	54 (35–66)	58 (42–69)	55 (38–66)
Age group	< 17 years	65 (8.7)	76 (11.2)	22 (7.1)	163 (9.4)
	**18–64 years**	482 (64.8)	396 (28.4)	181 (58.8)	**1059 (61.3)**
	≥ 65 years	196 (26.3)	192 (58.6)	103 (33.4)	491 (28.4)
	Not specified	1 (0.1)	12 (1.8)	2 (0.6)	15 (0.9)
Seriousness^b^	**Not serious**	549 (73.4)	431 (63.1)	180 (58.3)	**1160 (66.7)**
	Serious	199 (26.6)	252 (36.9)	129 (41.6)	580 (21.7)
	Other IME	103 (13.8)	183 (26.8)	91 (29.4)	377 (21.7)
	Hospitalization	85 (11.4)	49 (7.2)	29 (9.4)	163 (9.4)
	Life threatening	6 (0.8)	3 (0.4)	2 (0.6)	11 (0.6)
	Disabling	1 (0.1)	9 (1.3)	1 (0.3)	11 (0.6)
	Results in death	4 (0.5)	8 (1.2)	6 (1.9)	18 (1.0)
Outcome^b^	**Recovered/resolved**	318 (42.5)	390 (57.1)	107 (34.6)	**815 (46.8)**
	Recovering/resolving	318 (42.5)	103 (15.1)	85 (27.5)	506 (29.1)
	Not recovered/resolved	79 (10.69	124 (18.2)	66 (21.4)	269 (15.5)
	Recovered with sequelae	2 (0.3)	12 (1.8)	12 (3.9)	26 (1.5)
	Fatal	4 (0.5)	6 (0.9)	6 (1.9)	16 (0.9)
	Unknown	27 (3.6)	48 (7.0)	33 (10.7)	108 (6.2)
Source^b^	**Physician/doctor**	613 (82.0)	513 (75.1)	169 (54.7)	**1295 (74.4)**
	Patient/non-HCP	35 (4.7)	82 (12.0)	88 (28.5)	205 (11.8)
	Pharmacist	36 (4.8)	79 (11.6)	42 (13.6)	157 (9.0)
	Nurse	35 (4.7)	4 (0.6)	4 (1.3)	43 (2.5)
	Other HCP	29 (3.8)	5 (0.7)	6 (1.9)	40 (2.3)
	Lawyer	-	-	-	-
Suspected medicine	**Drug**	700 (93.6)	651 (95.3)	238 (77.0)	**2075 (90.5)**
	Vaccine	48 (6.4)	32 (4.7)	71 (23.0)	217 (9.5)

^a^ O-AEs codified with preferred terms of interest reported in our modified preferred term list (Table S1)

^b^ Of total received ICSRs

*HCP*: healthcare professional, *IQR*: interquartile range, *IME*: Important Medical Event

Most of the ICSRs referred to patients aged between 18 and 64 years (*n* = 1059; 61.3%), with a median age of 55 years (IQR = 38–66 years) and female (*n* = 1143; 65.7%) (Table 1). The majority of ICSRs were reported as not serious (*n* = 1160; 66.7%). A total of 580 (21.7%) safety cases met seriousness criteria. The most reported seriousness criterion was other medically important condition (*n* = 377; 21.7%), followed by caused/prolonged hospitalization (*n* = 163; 9.4%), resulted in death (*n* = 18; 1.0%), life-threatening (*n* = 11; 0.6%), and disabling (*n* = 11; 0.6%).

The outcome was reported for 1632 (93.8%) ICSRs and was mainly positive (recovered/resolved or recovering/resolving; *n* = 1321; 75.9%). Only a small percentage of safety cases (*n* = 16; 0.9%) had a fatal outcome.

The physician/doctor was the most frequent reporting source of the selected ICSRs (*n* = 1295; 74.4%), followed by the patient/non-HCPs (*n* = 205; 11.8%), the pharmacist (*n* = 15; 9.0%), by the nurse (*n* = 43; 2.5%), and by other HCPs (*n* = 40; 2.3%).

The ICSRs related to drugs (*n* = 2075; 90.5%) were largely more than those related to vaccines (*n* = 217; 9.5%). All characteristics of ICSRs for each Region are reported in Table 1.

### Comparison of the ICSRs across two time periods (2019–2021 vs. 2022–2024)

We observed similar characteristics in the ICSRs included in our analysis when compared to the findings from a previous analogous analysis covering the 2019–2021 period [4]. In both time periods, reports involved more women than men, a majority of individuals aged 18–64 years, predominantly non-serious AEs, and mostly favorable outcomes (i.e., recovered/resolved or recovering/resolving).

**Fig. 3:**
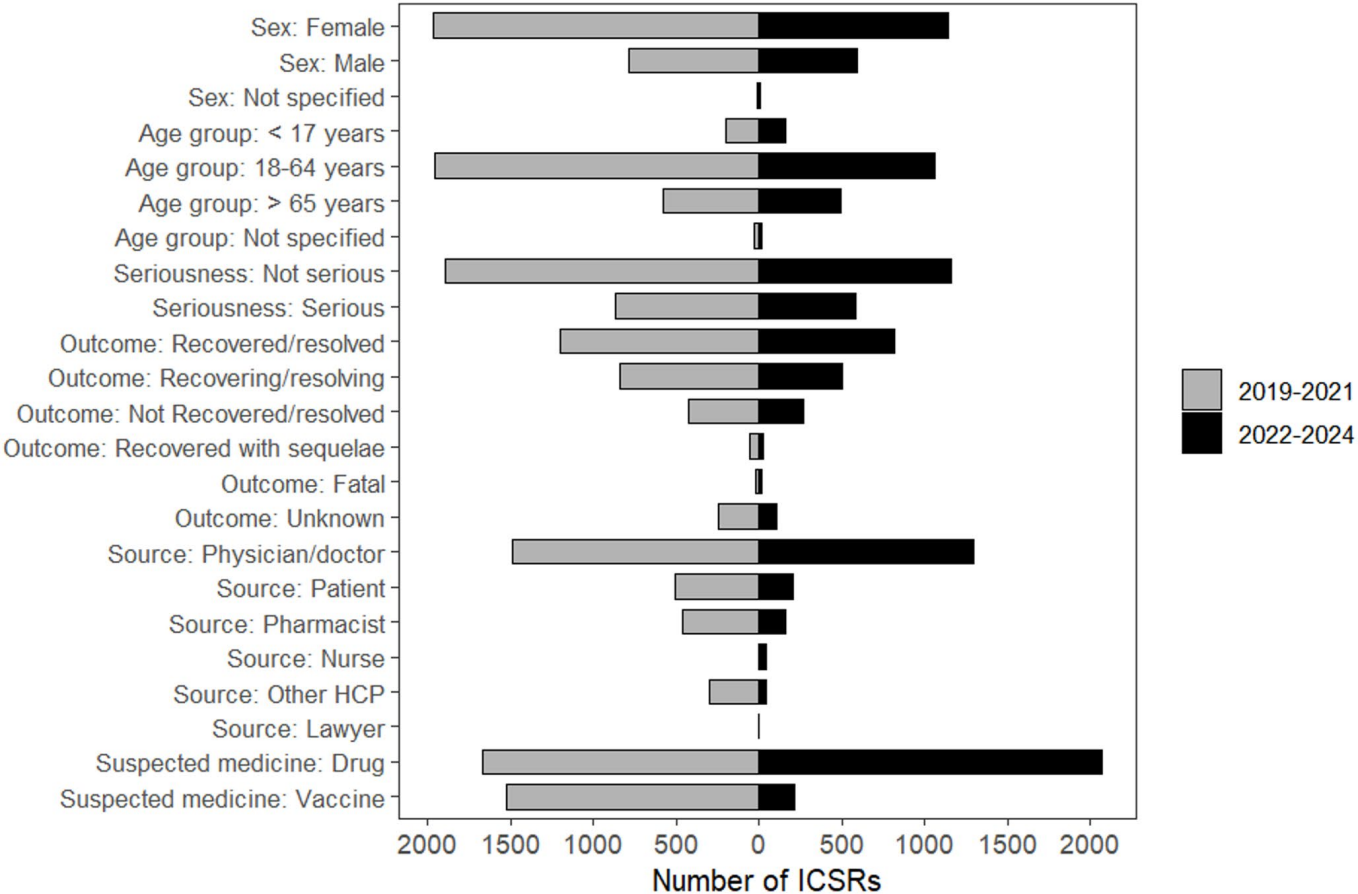
Distribution of Individual Case Safety Reports (ICSRs) collected in the Italian national pharmacovigilance network (*rete nazionale di farmacovigilanza*, RNF) with oropharyngeal adverse events for the periods 2019–2021 (left, in light grey) and 2022–2024 (right, in black), across different demographic, clinical, and reporting characteristics. Data for the 2019–2021 period have already been discussed and published by Sportiello et al. [4]. *HCP* healthcare professional

The only notable difference between the two time periods concerned the drug-to-vaccine ratio: in 2019–2021, vaccines were suspected medicines in 47.8% of reports, whereas in the following three-year period, this proportion decreased to 9.5% (Fig. 3).

Another notable difference concerned the prominence of MRONJ among the reported PTs. While ONJ was already included in the top twenty during 2019–2021, it rose in rank in 2022–2024, becoming the sixth most frequently reported PT (Table S2).

### Characteristics of O-AEs

**Fig. 4:**
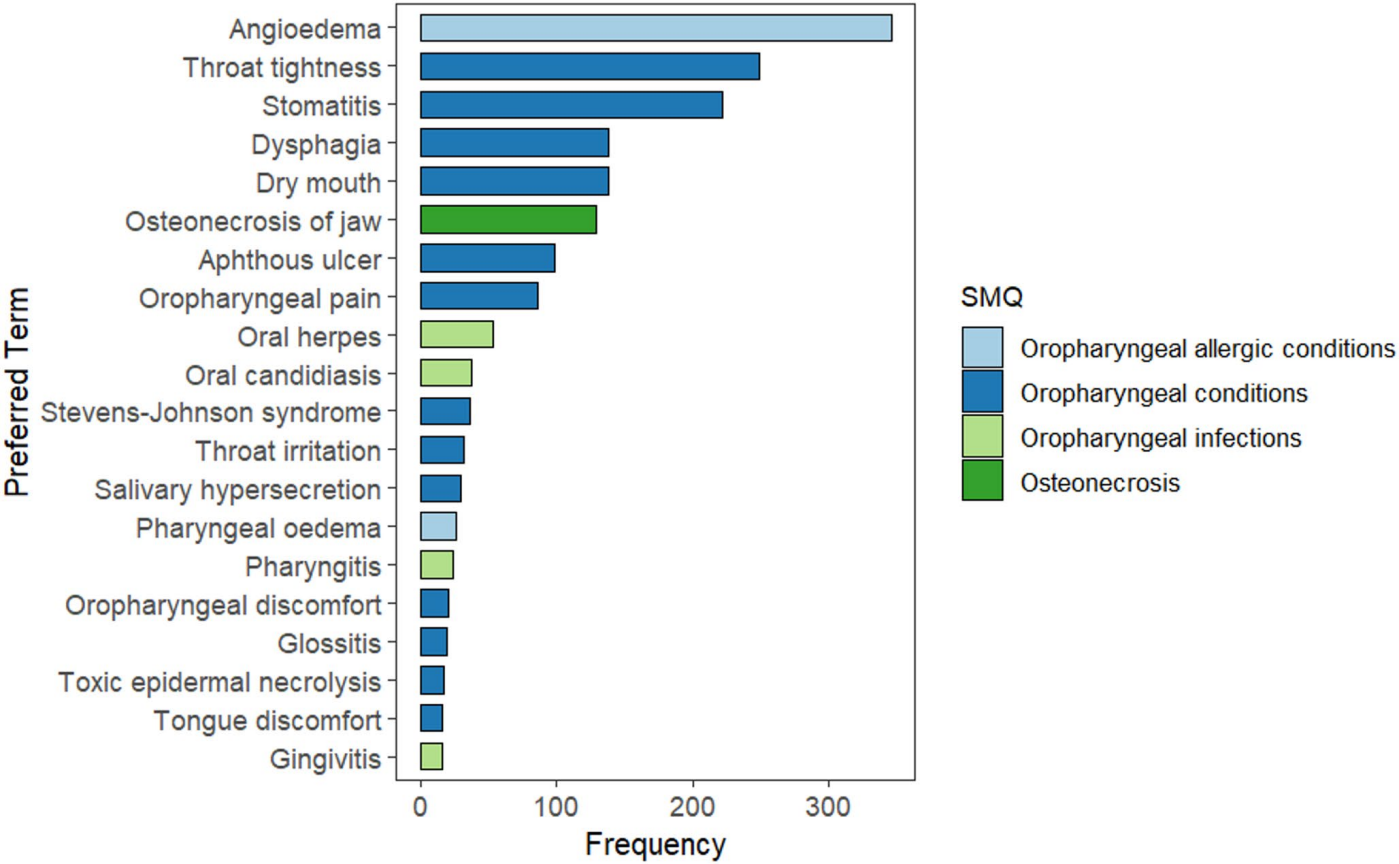
“Top twenty” preferred terms (PTs) reported in the individual case safety reports (ICSRs) containing at least one oropharyngeal adverse event (O-AEs) collected in the Italian national pharmacovigilance network (*rete nazionale di farmacovigilanza*, RNF) from 2022 to 2024 by three Italian regions (i.e., Piedmont, Campania, and Sicily)

The overall 1740 ICSRs accounted for a total of 1882 reported O-AEs (2 median O-AEs per ICSR; IQR: 1–4). Taking account that the distribution of AEs was categorized by SMQs, the most common SMQ was “oropharyngeal conditions (excluding neoplasms, infections and allergies)” (*n* = 1214; 64.5%), followed by “oropharyngeal allergic conditions” (*n* = 388; 20.6%), “oropharyngeal infections” (*n* = 134; 7.1%), “gingival disorders” (*n* = 5; 0.3%), and “oropharyngeal neoplasms” (*n* = 3; 0.2%) (Table 2). Analyzing data by each SMQ, more than half of AEs (61.6%) included in the first SMQ were throat tightness, stomatitis, dry mouth, and dysphagia. The 95.9% of the AEs related to the second SMQ “oropharyngeal allergic conditions” were angioedema and pharyngeal oedema. The first reported AEs in the third SMQ “oropharyngeal infections” were MRONJ, which covered 65.9% of the AEs related to this SMQ. The other SMQs – “gingival disorders” and “oropharyngeal neoplasms” – were involved for less than 1%. In Fig. 4, we reported the “top twenty” AEs coded as PT.

**Table 2:** Distribution of the first five preferred terms (PTs) for each standardized MedDRA query (SMQ) in the Individual Case Safety Reports (ICSRs) containing at least one oropharyngeal adverse event (O-AE, collected in the Italian national pharmacovigilance network (*rete nazionale di farmacovigilanza*, RNF) from 2022 to 2024 by three Italian regions (i.e., Piedmont, Campania, and Sicily)

Standardized MedDRA queries	Preferred term	Number of PTs (%)
Oropharyngeal conditions (1214; 64.5%)	Throat tightness	249 (20.5)
	Stomatitis	222 (18.3)
	Dry mouth	138 (11.4)
	Dysphagia	138 (11.4)
	Aphthous ulcer	99 (8.2)
	Oropharyngeal pain	86 (7.1)
Oropharyngeal allergic conditions (388; 20.6%)	Angioedema	346 (89.2)
	Pharyngeal oedema	26 (6.7)
	Mouth swelling	7 (1.8)
	Oropharyngeal swelling	5 (1.3)
	Gingival swelling	4 (1.0)
Oropharyngeal infections (138; 7.3%)	Oral herpes	53 (38.4)
	Oral candidiasis	38 (27.5)
	Pharyngitis	24 (17.4)
	Gingivitis	16 (11.6)
	Pharyngotonsillitis	3 (2.2)
Osteonecrosis (134; 7.1%)	Osteonecrosis of the jaw	129 (96.3)
	Osteonecrosis	4 (3.0)
	Tooth infection	1 (0.7)
Gingival disorders (5; 0.3%)	Noninfective gingivitis	3 (60.0)
	Gingival injury	1 (20.0)
	Gingivitis ulcerative	1 (20.0)
Oropharyngeal neoplasms (3; 0.2%)	Gingival cyst	2 (66.7)
	Squamous cell carcinoma of the oral cavity	1 (33.3)

A comparative analysis of the two study periods showed a largely stable distribution of O-AEs, with oropharyngeal conditions remaining the most frequently reported SMQ (65.9% in 2019–2021 vs. 64.5% in 2022–2024). Oropharyngeal allergic conditions increased (13.6% vs. 20.6%), whereas osteonecrosis and gingival disorders decreased (10.9% vs. 7.1% and 3.2% vs. 0.3%, respectively). At the PT level, throat tightness remained among the most frequently reported events and increased over time, while stomatitis and aphthous ulcer emerged prominently in 2022–2024. Angioedema showed a marked increase among allergic conditions, and osteonecrosis of the jaw became the predominant PT within the osteonecrosis SMQ (Table 3).

**Table 3:** Comparison of reported oropharyngeal adverse events (Preferred Terms) between 2019–2021 and 2022–2024 collected in the Italian national pharmacovigilance network (*rete nazionale di farmacovigilanza*, RNF) by three Italian regions (i.e., Piedmont, Campania, and Sicily). Data for the 2019–2021 period have already been discussed and published by Sportiello et al. [4]

Preferred terms	2019–2021	2022–2024	Change (percentage point)
**Oropharyngeal conditions**	2192 (100.0)	1240 (100.0)	
Throat tightness	266 (12.1)	249 (20.5)	+ 8.4
Stomatitis	0	222 (18.3)	+ 18.3
Dry mouth	177 (8.1)	138 (11.4)	+ 3.3
Dysphagia	181 (8.3)	138 (11.4)	+ 3.1
Aphthous ulcer	0	99 (8.2)	+ 8.2
Oropharyngeal pain	276 (12.6)	86 (7.1)	-5.5
Paresthesia oral	256 (11.7)	0	-11.7
**Oropharyngeal allergic conditions**	**453 (100.0)**	**388 (100.0)**	
Angioedema	166 (36.6)	346 (89.2)	+ 52.6
Pharyngeal oedema	29 (6.4)	26 (6.7)	+ 0.3
Mouth swelling	0	7 (1.8)	+ 1.8
Oropharyngeal swelling	0	5 (1.3)	+ 1.3
Gingival swelling	0	4 (1.0)	+ 1.0
Tongue edema	74 (16.3)	0	-16.3
Selling of tongue	71 (15.7)	0	-15.7
Palatal edema	47 (10.4)	0	-10.4
Oropharyngeal edema	29 (6.4)	0	-6.4
**Oropharyngeal infections**	**207 (100.0)**	**138 (100.0)**	
Oral herpes	109 (52.7)	53 (38.4)	-14.3
Oral candidiasis	14 (6.8)	38 (27.5)	+ 20.7
Pharyngitis	27 (13.0)	24 (17.4)	+ 4.4
Gingivitis	29 (14.0)	16 (11.6)	-2.4
Pharyngotonsillitis	0	3 (2.2)	+ 2.2
Tonsillitis	6 (2.9)	0	-2.9
**Osteonecrosis**	**362 (100.0)**	**134 (100.0)**	
Osteonecrosis of the jaw	95 (26.2)	129 (96.3)	+ 70.1
Osteonecrosis	28 (7.7)	4 (3.0)	-4.7
Tooth infection	0	1 (0.7)	+ 0.7
Bone pain	177 (48.9)	0	-48.9
Jaw pain	20 (5.5)	0	-5.5
Dental abscess	17 (4.7)	0	-4.7
**Gingival disorders**	**106 (100.0)**	**5 (100.0)**	
Noninfective gingivitis	9 (8.5)	3 (60.0)	+ 51.5
Gingival injury	0	1 (20.0)	+ 20.0
Gingivitis ulcerative	0	1 (20.0)	+ 20.0
Gingival bleeding	67 (63.2)	0	-63.2
Gingival pain	10 (9.4)	0	-9.4
Gingival hypertrophy	5 (4.7)	0	-4.7
Gingival discomfort	4 (3.8)	0	-3.8
**Oropharyngeal neoplasms**	**4 (100.0)**	**3 (100.0)**	
Gingival cyst	0	2 (66.7)	+ 66.7
Squamous cell carcinoma of the oral cavity	0	1 (33.3)	+ 33.3
Leukoplakia oral	2 (50.0)	0	-50.0
Oral neoplasm	1 (25.0)	0	-25.0
Oral papilloma	1 (25.0)	0	-25.0

Focusing on the cases of MRONJ, we found 129 cases. The majority of the reports involved female patients (*n* = 96, 74.4%), with a median patient age of 68 years (IQR = 60–77). Most reports were spontaneous (*n* = 82, 63.6%), and the primary sources of reports were predominantly hospital physicians (*n* = 81, 62.8%). In terms of outcomes, 19 cases (14.7%) were classified as recovered/resolved, 21 (16.3%) as recovering/resolving, 50 (38.8%) as not recovered/not resolved, and 15 (11.6%) as recovered with sequelae. No fatal outcomes were reported. Regarding seriousness, the majority of ICSRs (*n* = 107, 82.9%) were classified as serious due to other medically important conditions (Table 4). The ICSRs that reported MRONJ as an AE included 160 suspected drugs. The most frequently reported suspected drug was denosumab (*n* = 71, 44.4%), followed by zoledronic acid (*n* = 25, 15.6%), alendronic acid (*n* = 20, 12.5%), ibandronic acid (*n* = 14, 8.8%), and clodronic acid (*n* = 8, 5.0%) (Table 5).

**Table 4:** Characteristics of Individual Case Safety Reports (ICSRs) containing osteonecrosis of the jaw as an adverse event collected in the Italian national pharmacovigilance network (*rete nazionale di farmacovigilanza*, RNF) from 2022 to 2024 by three Italian regions (i.e., Piedmont, Campania, and Sicily)

Number of ICSRs (*N*, %)	Overall *N* = 129 (100.0)
**Sex**	
Female	96 (74.4)
Male	33 (25.6)
**Age years** (median [IQR])	69 (60–77)
**Report type**	
Spontaneous	82 (63.6)
Study report	47 (36.4)
**Primary source**	
Hospital physician	81 (62.8)
Physician	30 (23.3)
HP	11 (8.5)
Pharmacist	4 (3.1)
General physician	1 (0.8)
Patient	1 (0.8)
Local health authority professional	1 (0.8)
**Outcome**	
Recovered/resolved	19 (14.7)
Recovering/resolving	21 (16.3)
Not recovered/not resolved	50 (38.8)
Recovered with sequelae	15 (11.6)
Fatal	0
Unknown	24 (18.6)
**Seriousness**	
Not serious	6 (4.7)
Serious – other medically important condition	107 (82.9)
Serious – caused/prolonged hospitalization	6 (4.7)
Serious – disabling	10 (7.8)

**Table 5:** Distribution of suspected drugs reported in the individual case safety reports (ICSRs) related to osteonecrosis of the jaw, collected in the Italian national pharmacovigilance network (*rete nazionale di farmacovigilanza*, RNF) from 2022 to 2024 by three Italian regions (i.e., Piedmont, Campania, and Sicily)

Suspected drug	*n*	*N* (%)
Denosumab	71	71 (44.4%)
Zolendronic acid	25	25 (15.6%)
Alendronic acid	20	20 (12.5)
Ibandronic acid	14	14 (8.8)
Clodronic acid	8	8 (5.0)
Bevacizumab	4	4 (2.5)
Cabozantinib	4	4 (2.5)
Pembrolizumab	4	4 (2.5)
Everolimus	2	2 (1.3)
Lenvatinib	2	2 (1.3)
Adalimumab	1	1 (0.6)
Aflibercept	1	1 (0.6)
Guselkumab	1	1 (0.6)
Pamidronic acid	1	1 (0.6)
Risendronic acid	1	1 (0.6)
Rivaroxaban	1	1 (0.6)

### Distribution of suspected drugs or vaccines reported in the selected ICSRs

Considering the distribution of suspected medicines grouped by therapeutic group (first level of anatomical therapeutic chemical [ATC]), we observed that the most common reported level ATC groups were “L—antineoplastic and immunomodulating agents”(n = 1072; 46.8%), followed by “J–anti-infectives for systemic use” (n = 433; 18.9%), “M–musculoskeletal system” (n = 361; 15.8%), “N –nervous system” (n = 106; 4.6%), “A–alimentary tract and metabolism” (n = 62; 2.7%), “B–blood and blood forming organs” (n = 57; 2.5%), “C–cardiovascular system” (n = 56; 2.4%), and “R–respiratory system” (n = 50; 2.2%) (Table 6). The other ATC groups individually covered < 2%. The ATC group L was mainly characterized by the second-level ATC groups “L01–antineoplastic agents” (n = 874, 38.1%) and “L04–immunosuppressants” (n = 185; 8.1%); the second ATC group J by “J07–vaccines” (n = 217; 9.5%) and “J01–antibacterials for systemic use” (n = 197; 8.6%) (Table 6). Among vaccine-related ICSRs, the vast majority were associated with COVID-19 vaccines, whereas reports involving other vaccines were limited (data not shown).

**Table 6:** Distribution of suspected medicinal products classified by therapeutic group (second level-anatomical therapeutic chemical, ATC) collected in the Italian national pharmacovigilance network (*rete nazionale di farmacovigilanza*, RNF) from 2022 to 2024 by three Italian regions (i.e., Piedmont, Campania, and Sicily)

ATC – level	*N* (%)
L – Antineoplastic and immunomodulating agents	1072 (46.8)
L01 antineoplastic agents	874 (38.1)
L04 immunosuppressants	185 (8.1)
L02 endocrine therapy	9 (0.4)
L03 immunostimulants	4 (0.2)
J – Anti-infective for systemic use	433 (18.9)
J07 vaccines	217 (9.5)
J01 antibacterials for systemic use	197 (8.6)
J05 antivirals for systemic use	12 (0.5)
J02 antimycotics for systemic use	4 (0.2)
J04 antimycobacterials	3 (0.1)
M – Musculoskeletal system	361 (15.8)
M01 anti-inflammatory and antirheumatic products	169 (7.4)
M05 drugs for treatment of bone disease	146 (6.4)
M04 antigout preparations	35 (1.5)
M03 muscle relaxants	8 (0.3)
M02 topical products for joint and muscular pain	3 (0.1)
N – Nervous system	106 (4.6)
N02 analgesics	48 (2.1)
N05 psycholeptics	27 (1.2)
N06 psychoanaleptics	16 (0.7)
N03 antiepileptics	10 (0.4)
N01 anesthetics	5 (0.2)
A – Alimentary tract and metabolism	62 (2.7)
A02 drugs for acid-related disorders	15 (0.7)
A10 drugs used in diabetes	13 (0.6)
A03 drugs for functional gastrointestinal disorders	10 (0.4)
A04 antiemetics and antinauseants	8 (0.3)
A07 antidiarrheals, intestinal anti-inflammatory/anti-infective agents	6 (0.3)
A01 stomatological preparations	3 (0.1)
A12 mineral supplements	3 (0.1)
A06 drugs for constipation	1 (< 0.1)
A11 vitamins	1 (< 0.1)
A16 other alimentary tract and metabolism products	1 (< 0.1)
A05 bile and liver therapy	1 (< 0.1)
B – Blood and blood forming organs	57 (2.5%)
B01 – antithrombotic agents	30 (1.3)
B03 – antianemic preparations	25 (1.1)
B06 – other hematological agents	1 (< 0.1)
B02 – antihemorrhagics	1 (< 0.1)
C – Cardiovascular system	56 (2.4)
C09 – agents acting on the renin-angiotensin system	18 (0.8)
C10 – lipid modifying agents	15 (0.7)
C07 – beta blocking agents	9 (0.4)
C08 – calcium channel blockers	3 (0.1)
C03 – diuretics	3 (0.1)
C01 – cardiac therapy	3 (0.1)
C05 – vasoprotectives	2 (< 0.1)
C02 – antihypertensives	2 (< 0.1)
C04 – peripheral vasodilators	1 (< 0.1)
R – Respiratory system	50 (2.2)
R03 – drugs for obstructive airway diseases	27 (1.2)
R06 – antihistamines for systemic use	15 (0.7)
R05 – cough and cold preparations	3 (0.1)
R02 – throat preparations	3 (0.1)
R01 – nasal preparations	2 (< 0.1)
V – Various	34 (1.5)
V08 –contrast media	27 (1.2)
V03 – all other therapeutic products	4 (0.2)
V01 – allergens	3 (0.1)
H – Systematic hormonal preparations, excl. sex hormones and insulins	28 (1.2)
H02 – corticosteroids for systematic use	19 (0.8)
H05 – calcium homeostasis	4 (0.2)
H01 – pituitary and hypothalamic hormones and analogues	3 (0.1)
H03 – thyroid therapy	3 (0.1)
D – Dermatologicals	12 (0.5)
D11 – other dermatological preparations	6 (0.3)
D07 – corticosteroids, dermatological preparations	4 (0.2)
D06 – antibiotics and chemotherapeutics for dermatological use	1 (< 0.1)
D08 – antiseptics and disinfectants	1 (< 0.1)
G – Genitourinary system and sex hormones	9 (0.4)
G04 – urologicals	6 (0.3)
G03 – sex hormones and modulators of the genital system	3 (0.1)
S – Sensory organs	7 (0.3)
S01 – ophthalmologicals	7 (0.3)
P – Antiparasitic products, insecticides and repellents	4 (0.2)
P01 – antiprotozoals	4 (0.2)

### Disproportionality analysis

Looking at the probability of reporting O-AEs by each source, physicians/doctors were associated with a lower probability of reporting these events compared to patients/non-HCPs (ROR: 0.72, 95% CI 0.62–0.84) and nurses (ROR: 1.34, 95% CI 0.98–1.87), while no statistically significant associations were observed comparing physician/doctors with pharmacists and other HCPs. Moreover, patients/non-HCPs were also associated with a higher probability of reporting O-AEs when compared to nurses (ROR: 1.86, 95% CI 1.32–2.66) (Table 7).

**Table 7:** Disproportionality analysis (Reporting Odds Ratio [ROR] with 95% Confidence Interval [CI]) by source of reporting, based on individual case safety reports (ICSRs) collected in the Italian national pharmacovigilance network (*rete nazionale di farmacovigilanza*, RNF) from 2022 to 2024 by three Italian regions (i.e., Piedmont, Campania, and Sicily)

Comparison	ROR (95% CI)	*p*-value
*Physician/doctor*		
vs. patient/non-HCP	0.72 (0.62–0.84)	<<0.05
vs. pharmacist	0.78 (0.66–0.93)	< 0.05
vs. nurse	1.34 (0.98–1.87)	0.06
vs. other HCP	0.84 (0.61–1.19)	0.29
*Other HCP*		
vs. oatient/non-HCP	0.86 (0.59–1.22)	0.45
vs. pharmacist	0.94 (0.64–1.34)	0.79
vs. nurse	1.60 (1.00–2.54)	0.04
*Patient/non-HCP*		
vs. pharmacist	1.09 (0.88–1.36)	0.45
vs. nurse	1.86 (1.32–2.66)	< 0.05
*Nurse*		
vs. pharmacist	0.58 (0.40–0.83)	< 0.05

*HCP* healthcare professional

In our study, the majority of physicians who reported ICSRs with O-AEs were hospital physicians (*n* = 974; 75.2%), while general practitioners accounted for only 5.5% (*n* = 72) (data not shown).

## Discussion

In this study, we investigated O-AEs through the analysis of post-marketing safety data from the RNF of three regions of Italy – Campania, Sicily, and Piedmont – in a limited period (2022–2024). Our analysis included a total of 1740 ICSRs reporting O-AEs, accounting for less than 4% of all ICSRs reported in the same period and geographic areas. Although the proportion appears relatively low, O-AEs represent a clinically relevant subset of AEs due to their potential impact on patient compliance and quality of life [4].

Moreover, these events are likely underreported, primarily due to challenges in clinical recognition and limited adherence to established procedures, compounded by frequent underreporting within pharmacovigilance practices among HCPs [8].

In the first months of 2022, most of the ICSRs concerned vaccines, particularly those against COVID-19. This data is in line with the epidemiological and health context of the period, characterized by an intense vaccination campaign and particularly high media attention towards vaccine safety [14, 15]. In this scenario, there was likely a greater propensity for reporting from both HCP sand citizens, from the perspective of enhanced pharmacovigilance promoted by regulatory authorities [16]. However, starting from 2023, a reversal of the trend was observed, with a reduction in the number of ICSRs reported for vaccines (only 7 in 2023, representing 1.1%, and 8 in 2024, representing 1.3%). This data can be attributed to several factors. Firstly, the reduction in the number of vaccine administrations in the general population has inevitably led to a decrease in reports [17]. Secondly, the reduced level of media attention may have decreased the sensitivity of patients and HCPs in relation to the spontaneous report [17]. Furthermore, it should be considered that, over time, the safety profile of COVID-19 vaccines has been confirmed to be favorable, and this has likely led to less propensity to report spontaneously, especially in the absence of new or unexpected AEs. Most of the ICSRs concerned female patients aged between 18 and 64 years. This data is consistent with post-marketing data, which show a higher susceptibility and severity to experiencing AEs in women compared to men [18]. Moreover, the high number of cases in the age group 18–64 reflects the greater pharmacological exposure, polytherapy, and sensitivity of the oral mucosa in this age group [19]. In our study, most of the ICSRs were classified as “not serious”. Generally, the O-AEs reported in pharmacovigilance studies (e.g., dry mouth, dysgeusia, stomatitis, oral burning, localized ulcers) are usually mild or moderate and reversible after discontinuation of treatment [20]. Furthermore, those events rarely require hospitalization, urgent medical interventions, or permanent disability. However, serious O-AEs have been reported after antipsychotic administration [21]. In line with other pharmacovigilance studies, the main source of reporting is represented by the HP [22, 23]. They have the necessary skills to recognize signs and symptoms potentially associated with the use of medications, even when it comes to non-severe or nonspecific manifestations, such as oral ones (e.g., xerostomia, dysgeusia, stomatitis) [20]. Moreover, at the regulatory level, their involvement in pharmacovigilance activities is strongly encouraged by the ministerial decree of April 30, 2015, aimed at ensuring continuous monitoring of drug safety in clinical practice. The majority of the ICSRs were related to drugs (*n* = 2075; 90.5%), while 9.5% were related to vaccines. These data highlight a reversal compared to the previous pandemic three-year period (2020–2022), during which a peak of reports associated with vaccines was observed, particularly with the anti-COVID-19 vaccines [4]. Our results highlight a return to spontaneous reporting from drugs consistent with their wider and ongoing exposure in the population. Despite this shift in the distribution of suspected products, the overall clinical pattern of reported O-AEs remained largely consistent with that observed in the 2019–2021 period. In both study periods, oropharyngeal conditions represented the most frequently reported SMQ. However, some changes emerged over time, including an increase in oropharyngeal allergic conditions (primarily driven by angioedema) and a decrease in osteonecrosis and gingival disorders. These findings suggest substantial stability in the overall spectrum of O-AEs, while also reflecting temporal changes in the relative contribution of specific clinical presentations.

The most frequently observed SMQs were “oropharyngeal conditions”, “oropharyngeal allergic conditions” and “oropharyngeal infections”, highlighting symptoms and conditions sustained by pathogens (e.g., oral herpes), attributable to allergic reactions (e.g., angioedema) or that do not present a clear infectious or allergic etiology (e.g., throat tightness). Analyzing the individual AEs, those reported most frequently were angioedema, throat tightness, stomatitis, dry mouth, and dysphagia [24]. Angioedema is well documented in the literature as anAE resulting from the use of drugs such as antimicrobials, angiotensin-converting enzyme (ACE) inhibitors, and nonsteroidal anti-inflammatory drugs (NSAIDs) [20, 25, 26]. Throat tightness is often described as a symptom of anaphylaxis [27]. In some cases, throat constriction along with coughing can precede respiratory distress due to the onset of oropharyngeal or laryngeal edema with dyspnea, hoarseness, stridor, and even aphonia, or related to bronchospasm with tachypnea and wheezing [27]. Stomatitis, also known as oral mucositis, represents an inflammatory process that can be caused by a variety of causes such as infections (e.g., viral or bacterial), allergic reaction, systemic disease (e.g., Behçet’s syndrome), nutritional deficiencies (e.g., vitamin B12), or physical/chemical irritants [28–30]. The risk factors that could influence the development of mucositis can vary, such as age of patients, sex, the presence of dental appliances, a history of oral lesions, or smoking [31]. However, it is one of the main complications manifested by about 30% to 40%of patients undergoing chemotherapy treatment that can cause reduced proliferation of basal epithelial cells, resulting in thinning of the superficial epithelium and the onset of mucositis [28, 32, 33]. Dry mouth is one of the O-AE most common symptoms associated with medications (e.g., tricyclic antidepressants, antipsychotics, beta-blockers). In most cases, it is reversible, and it is due to an anticholinergic or sympathomimetic action [20]. Dysphagia manifests in 8% of the population, and its prevalence is estimated to increase in the older adult population [34]. Drug-induced dysphagia can lead to physiological dysfunction and is mainly associated with skeletal muscle relaxants, antipsychotics, dementia drugs, sedative-hypnotics/anti-anxiety medications, and antiepileptics [35–38]. Our results showed that, in the three years, 2022–2024, most reports of O-AEs were associated with drugs belonging to the ATC class L (i.e., antineoplastic agents) and J07 (i.e., vaccines). Antineoplastic drugs and immunomodulators are known to cause reactions affecting the oral cavity, particularly mucositis, stomatitis, ulcers, and alterations in taste [39–41]. These arise mainly from the direct cytotoxic action on highly proliferative epithelial cells [42]. The introduction in recent years of new oncological drugs such as checkpoint inhibitors seems to have increased the frequency of oral immune-related AEs (> 20%), leading health operators to observe a new toxicity profile for these drugs [43]. Indeed, such manifestations differ from those observed with cytotoxic chemotherapy and/or traditional radiotherapy and can lead to significant morbidity or treatment interruption [43].

During the study period, we observed that 129 ICSRs reported osteonecrosis of the jaw, a number considerably lower than the MRONJ cases documented in theItalian literature [44–51]. This reduction in reported cases may, at least in part, reflect the impact of regulatory risk minimization measures implemented over the past years to mitigate the occurrence of MRONJ, such as the inclusion of specific warnings in the Summary of Product Characteristics (SmPC), patient information leaflets, the introduction of patient reminder cards, and direct healthcare professional communication (DHPC) [52]. Most of them were related to female subjects (74.4%) aged between 60 and 77 years, consistent with other pharmacovigilance studies [53–55]. MRONJ is a severe AE experienced by some patients to certain drugs commonly used in the treatment of cancer and osteoporosis. Depending on the type and dosing of drugs, this AE may occur rarely (e.g., following the oral administration of bisphosphonate or denosumab treatments for osteoporosis, or antiangiogenic agent-targeted cancer treatment), or commonly (e.g., following intravenous bisphosphonate for cancer treatment). MRONJ can greatly affect the quality of life of patients if not promptly diagnosed and treated. In our study, antiresorptive medications, including bisphosphonates and denosumab, were the major medications associated with this event. The pathophysiology of MRONJ induced by antiresorptive medications involves bone remodeling inhibition and angiogenesis inhibition. Our findings should also be interpreted in light of the well-recognized limitations of spontaneous reporting systems, particularly for adverse events such as MRONJ. As highlighted by Edwards et al., the identification of bisphosphonate-related osteonecrosis of the jaw initially relied on spontaneous case reports, which were instrumental in signal detection, but substantially underestimated the true burden of the condition [56]. Subsequent prospective and observational studies provided more reliable incidence estimates, often considerably higher than those derived from spontaneous reporting databases. In particular, prospective cohort studies have shown that the cumulative incidence of MRONJ increases with treatment duration and varies according to the specific antiresorptive agent and underlying indication.

Finally, our disproportionality analysis, which assesses the relative likelihood of reporting O-AEs rather than the absolute number of reports, revealed a significantly lower likelihood of physicians reporting O-AEs compared to both patients/non-healthcare professionals and pharmacists. Although the comparison with nurses did not reach conventional statistical significance, the direction of the association suggests that nurses may be more likely to report these events than physicians. These findings indicate that physicians might underreport O-AEs relative to other reporter groups. Several factors could explain this discrepancy, including differences in clinical priorities, underestimation of the clinical relevance of oropharyngeal symptoms, or time constraints in routine practice [57]. Conversely, patients and nurses may be more attentive to local mucosal symptoms that affect daily comfort or oral function, thus more likely to perceive and report them. These observations highlight the need to promote awareness and reporting engagement among physicians, especially for O-AEs that may initially appear minor but can affect adherence and patient quality of life.

This issue of underreporting by physicians parallels the low level of patient awareness regarding serious O-AEs, such as MRONJ. A recent systematic review highlighted that only 19% of patients under bone-modifying agents were aware of MRONJ risk, emphasizing the critical role of effective communication and interprofessional collaboration in improving preventive strategies [58]. This reinforces the need for a patient-centered approach where clinicians, pharmacists, and oral health professionals actively inform patients about potential oral ADRs to enhance adherence and early detection.

Similar concerns about underreporting and insufficient awareness have been documented in other healthcare settings. For instance, Coppini et al. highlighted that in aesthetic medicine and dental practice, the lack of a standardized pharmacovigilance culture and training significantly contributes to missed ADR notifications [59].

These findings align with our observation that even HPs may underestimate the relevance of oropharyngeal manifestations, reinforcing the need for targeted educational programs [60].

### Strengths and limitations

This study represents our second investigation in Italy focusing on O-AEs using post-marketing data from the RNF, specifically from the regions of Piedmont, Campania, and Sicily. Our previous analysis, which covered the 2019–2021 period, was significantly affected by the high volume of ICSRs related to COVID-19 vaccines, largely due to the mass vaccination campaign active during that time. This constituted a major limitation, as it potentially skewed the overall distribution and characterization of reported O-AEs. In the present study, this limitation was overcome by analyzing a more recent time frame (2022–2024), in which the impact of pandemic-related reporting was considerably reduced.

As in our previous study, the decision to focus on this specific safety concern stemmed from the need to explore in greater depth the real-world data on O-AEs. Investigating O-AEs within a post-marketing surveillance framework offers a valuable perspective to promote more rational drug use, guide clinical behavior, and ultimately enhance patient safety. This focus on real-life pharmacovigilance data remains one of the key strengths of our research.

Moreover, several study limitations should be taken into consideration. The results from our study are subject to typical pharmacovigilance biases, including under-reporting [61]. Additionally, the RNF does not routinely capture exposure denominators, such as the number of patients treated with specific medications at the regional level. Since the present project was specifically designed to rely exclusively on RNF data, crude reporting rates or incidence estimates could not be calculated. Therefore, our analyses were limited to descriptive and disproportionality approaches. Furthermore, as already discussed in our previous study, stimulated reporting may have influenced reporting patterns, particularly during the COVID-19 vaccination campaign, when heightened public awareness and media attention likely led to a temporary overrepresentation of vaccine-related reports [4].

### Conclusions

In conclusion, when compared to the findings from a previous analogous analysis, our results confirmed that a small percentage (3.6%) of ICSRs reported were related to O-AEs. The two analyses in comparison demonstrated similar characteristics in the ICSRs in terms of sex (the most reported as females), age (the most reported as 18–64 years), seriousness of AEs (the most reported as non-serious), and outcomes (the most reported as recovered/resolved and recovering/resolving). The influence of COVID-19, shown in the first period (2019–2021), has disappeared in the second period (2022–2024) following the reduction of vaccine campaigns and media attention. In fact, the reporting focused nearly exclusively on the drugs and not the vaccines. Additionally, the nature of O-AEs appears to result in a higher reporting frequency of non-serious cases, potentially reflecting their perceived milder presentation. We found 129 cases of MRONJ with an unfavorable evolution and mostly serious. In line with the literature, denosumab and bisphosphonates were the most reported drugs potentially responsible for these events. Another notable finding from the disproportionality analysis was that the probability of reporting oral AEs was less frequent with physicians than with patients or other healthcare professionals. Thus, there is a clear need to strengthen awareness and reporting efforts by physicians (mainly dentists), especially for O-AEs that may initially appear mild but later worsen or be correlated to a more serious pathological condition. Promoting a shared and inclusive pharmacovigilance culture among healthcare professionals is essential for enhancing patient safety and ensuring more timely, accurate, and consistent reporting of AEs.

## Supplementary Information

Below is the link to the electronic supplementary material.

Supplementary Material 1

## Data Availability

The use of the raw data that support the findings of this study has been authorized by the Italian Medicines Agency (AIFA), which holds the ownership of the data. Data can be available with the permission of AIFA upon reasonable request.

## Abbreviations

ACE: Angiotensin-Converting Enzyme
AE: Adverse event
AEFI: Adverse event following immunization
ADR: Adverse drug reaction
AIFA: Agenzia Italiana del Farmaco
ATC: Anatomical Therapeutic Chemical
CI: Confidence interval
COVID-19: Coronavirus disease-19
DHPC: Direct healthcare professional communication
EMA: European Medicines Agency
EU: European Union
EV: EudraVigilance
FDA: Food and Drug Administration
HCP: Healthcare Professional
ICH: International Council for Harmonisation of technical requirements for pharmaceuticals for human use
ICSR: Individual Case Safety Reports
IME: Important Medical Event
IQR: Interquartile Range
LLT: Low- Level Term
MedDRA: Medical Dictionary for Regulatory Activities
MRONJ: Medication- related osteonecrosis of the jaw
NSAIDs: Nonsteroidal anti-inflammatory drugs
O-AE: Oropharyngeal Adverse Event
PT: Preferred Term
QPPV: Qualified Persons for Pharmacovigilance
RNF: Rete Nazionale di Farmacovigilanza
ROR: Reporting Odds Ratio
SMQ: Standardized MedDRA Query
SOC: System Organ Class
