# Supplementary material for: Italian regional data and oropharyngeal adverse events to drugs and vaccines: insights from a 3-year spontaneous reporting (2022–2024)
Source: Pharmacol Rep. 2026 Jun 15;78(4):1275–91. doi: 10.1007/s43440-026-00873-3 (PMC13437578; doi:10.1007/s43440-026-00873-3)
Supplement: Supplementary file 1 [file 43440_2026_873_MOESM1_ESM.docx]

**Table S1. Modified list of all Preferred Terms (PTs) divided in six Standardized MedDRA Query (SMQ) used to perform the data analysis.**

**Oropharyngeal disorders (SMQ)**

**Oropharyngeal allergic conditions (SMQ)**

Angioedema

Gingival swelling

Mouth oedema/edema

Swelling of tongue

Tongue oedema/edema

Gingival oedema/Edema gum

Palatal oedema/edema

Contact stomatitis

Idiopathic angioedema

Palatal swelling

Mouth swelling

Pharyngeal oedema

Epiglottic oedema/edema

Epiglottitis

Oropharyngeal swelling

Allergic stomatitis

**Oropharyngeal conditions (excluding neoplasms. infections and allergies) (SMQ)**

Aphthous ulcer

Aptyalism

Behcet syndrome

Buccoglossal syndrome

Dry mouth

Dry throat

Dysphagia

Foreign body in pharynx

Glossitis

Glossodynia

Tongue papillary hypertrophy NOS

Melkersson- Rosenthal syndrome

Mouth bleeding

Ulcer buccal

Muco-cutaneo-ocular syndrome

Odynophagia

Oral disorder

Oral lichen planus

Oral mucosa blister/blistering

Oral mucosal/mucosa discolouration

Oral mucosal eruption

Oral pain/pain oral

Oral papule

Oral submucosal fibrosis

Oropharyngeal spasm

Parotid duct obstruction

Parotid gland enlargement

Plicated tongue

Protrusion tongue

Ranula

Altered saliva

Salivary duct obstruction

Salivary duct stenosis

Salivary gland atrophy

Salivary gland calculus

Salivary gland enlargement

Salivary gland fistula

Salivary gland pain

Salivary hypersecretion

Sjögren's syndrome

Stevens-Johnson syndrome

Stomatitis

Stomatitis haemorrhagic

Stomatitis necrotising

Stomatitis radiation

Submaxillary gland enlargement

Throat irritation

Throat tightness

Tongue blistering

Coated tongue/Tongue coated

Tongue discoloration/Discoloration tongue

Tongue disorder/Tongue disorder NOS

Lingua geographica/Geographic tongue/Tongue geographic

Tongue hematoma/haematoma

Tongue movement disturbance

Tongue disorder

Tongue rough

Tongue spasm/Spasm tongue

Tongue ulceration

Tonsillar hypertrophy

Toxic epidermal necrolysis

Parotid lipomatosis

Sialectasia

Saliva discoloration

Ankyloglossia acquired

Laceration of mouth

Tongue dry

Tongue haemorrhage

Oral mucosa atrophy

Tonsillar ulcer

Glossodynia

Parotid gland haemorrhage/hemorrhage

Mikulicz's syndrome

Sialocele

Tonsillar atrophy

Saburral tongue

Uvulitis

Tongue eruption

Salivary gland cyst

Mikulicz's syndrome

Palatal disorder

Palatal dysplasia

Itching mouth

Tonsillar disorder

Throat lesion

Tongue atrophy

Lip laceration

Salivary duct inflammation

Salivary gland mass

Oral leukoedema

Hypoesthesia oral

Paresthesia oral

Tonsillar haemorrhage/hemorrhage

Oral toxicity

Acquired macroglossia

Glossoptosis

Auriculotemporal syndrome

Tongue laceration

Salivary gland mucocele/mucocoele

Salivary gland disorder/NOS

Foaming at mouth

Oral mucosal hypertrophy

Tonsillolith

Angina bullosa haemorrhagica/hemorrhagica

Oral mucosal exfoliation

Tongue exfoliation

Oral mucosa erosion

Oroantral fistula

Tonsillar inflammation

Oral cavity fistula

Sialometaplasia

Velopharyngeal incompetence

Tongue necrosis

Oral mucosal erythema

Oral disorder

Oropharyngeal plaque

Oropharyngeal blistering

Burning mouth syndrome

Oropharyngeal discomfort

Oropharyngeal pain

Radiation salivary gland injury

Atrophic glossitis

Tongue pigmentation

Tongue itching

PFAPA syndrome

Chemical burn of oral cavity

Oropharyngeal scar

Nicotinic stomatitis

Pharyngeal dyskinesia

Exposed bone in jaw

Salivary gland induration

Palatal palsy

Sialography abnormal

Pyostomatitis vegetans

Tongue infarction

Scalloped tongue

Oral mucosa haematoma/hematoma

Noninfective sialoadenitis

Acute postoperative sialadenitis

Coating in mouth

Burn oral cavity

Cobble stone tongue

Traumatic ulcerative granuloma with stromal eosinophilia

Oral hyperesthesia

Oral blood blister

Palatal ulcer

Oropharyngeal cobble stone mucosa

Oral pigmentation

Tongue discomfort

Chronic cheek biting

MAGIC syndrome

Oral contusion

Tonsillar erythema

Tonsillar exudate

Oropharyngeal oedema/edema

Tongue erythema

Oropharyngeal discolouration/discoloration

Transient lingual papillitis

Throat clearing

Trichoglossia

Cricopharyngeal achalasia

Pharyngeal dystonia

Stiff tongue

Palatal polyp

Lichenoid dysplasia

Tongue thrust

Anesthesia oral

Lip scab

Oral mucosal scab

Mucocutaneous disorder

SJS-TEN overlap

Oral macule

Foreign body in pharynx

Acute graft versus host disease oral

Chronic graft versus host disease oral

Oral purpura

Oral lichenoid reaction

Oral mucosal roughening

Oral mucosal scar

Tongue induration

Palatal prolapse

Oropharyngeal lymphoid hyperplasia

Acquired soft palate fissure

Orofacial granulomatosis

Uvula deviation

Oral vaccine administration site lesions

Pharyngeal hypoesthesia

Pharyngeal swelling

Pharyngeal erythema

Pharyngeal paresthesia

Diffuse infiltrative lymphocytosis syndrome

**Oropharyngeal infections (SMQ)**

Abscess of salivary gland

Abscess oral

Angina gangrenous

Aspergillosis oral

Chronic tonsillitis

Gingivitis

Hand. foot and mouth disease

Herpangina

Mumps

Oral candidiasis

Parotid abscess

Parotitis

Parotiditis

Abscess peritonsillar/NOS

Sialoadenitis/NOS

Tonsillitis/NOS

Streptococcal tonsillitis

Viral tonsillitis

Oral infection

Ludwig angina

Pharyngotonsillitis

Peritonsillitis

Oropharyngeal candidiasis

Strawberry tongue

Gingival abscess

Oro-pharyngeal aspergillosis

Necrotizing ulcerative gingivostomatitis

Necrotizing ulcerative periodontitis

Oral pustule

Oral fungal infection

Oropharyngitis fungal/NOS

Oral bacterial infection

Oral viral infection

Tonsillitis bacterial

Tonsillitis fungal

Oral helminthic infection

Lip infection

Oropharyngeal gonococcal infection

Tongue abscess

Oral herpes

Lineal gingival erythema

Staphylococcal parotitis

Pharyngeal abscess

Infective glossitis

Viral parotitis

Herpes simplex pharyngitis

Herpes zoster pharyngitis

Tongue fungal infection

Oral tuberculosis

Bacterial parotitis

Parapharyngeal space infection

Pharyngitis

Bacterial gingivitis

**Gingival disorders (SMQ)**

Gingival atrophy

Gingival bleeding/Bleeding gingival

Gingival discolouration/discoloration

Gingival disease

Erosion gingival

Gingival hypertrophy/Gum hypertrophy

Gingival hypoplasia/Gum hypoplasia

Gingival pain/gum pain/pain gum

Gingival/Gum recession

Gingivitis ulcerative

Periodontal destruction

Periodontal disease

Gingival injury

Gingival blister

Itching gum

Gingival/Gum ulceration

Gingival hyperpigmentation

Gingival erythema

Periodontal inflammation

Noninfective gingivitis

Gingival/Gum discomfort

Excessive gingival display

Gingival/Gum scar

Dental root perforation

**Oropharyngeal neoplasms (SMQ)**

Salivary gland neoplasm benign/NOS

Giant cell epulis

Gingival polyp

Leukoplakia oral/NOS

Lip and/or oral cavity cancer recurrent

Lip and/or oral cavity cancer stage 0

Lip and/or oral cavity cancer stage I

Lip and/or oral cavity cancer stage II

Lip and/or oral cavity cancer stage III

Lip and/or oral cavity cancer stage IV

Malignant palate neoplasm

Mouth cyst

Oral hairy leukoplakia

Oral neoplasm benign

Salivary gland cancer recurrent

Salivary gland cancer stage 0

Salivary gland cancer stage I

Salivary gland cancer stage II

Salivary gland cancer stage III

Salivary gland cancer stage IV

Squamous cell carcinoma of the oral cavity

Squamous cell carcinoma of the tongue

Tongue neoplasm benign

Malignant neoplasm of tongue. unspecified

Tonsillar neoplasm

Benign tonsillar neoplasm

Buccal polyp

Salivary gland adenoma

Tongue dysplasia

Oral fibroma

Metastases to mouth

Metastases to salivary gland

Tongue cyst

Gingival cyst

Oral cavity cancer metastatic

Tongue cancer metastatic

Tonsillar cyst

Epulis

Tongue carcinoma stage 0

Tongue carcinoma stage I

Tongue carcinoma stage II

Tongue carcinoma stage III

Tongue carcinoma stage IV

Oral neoplasm/NOS

Salivary gland neoplasm/cancer/NOS

Lip and/or oral cavity cancer

Salivary gland cancer

Leukoplakia/NOS

Tongue neoplasm/NOS

Tonsillar neoplasm/cancer/NOS

Metastatic salivary gland cancer

Gingival cancer

Melanoplakia oral

Adenocarcinoma of salivary gland

Oral papilloma

Tongue cancer recurrent

Tonsil cancer metastatic

Acinic cell carcinoma of salivary gland

Adenoid cystic carcinoma of salivary gland

Mucoepidermoid carcinoma of salivary gland

Pleomorphic adenoma

Papillary cystadenoma lymphomatosum

Tongue polyp

Carcinoma ex-pleomorphic adenoma

Metastases to tonsils

Oral haemangioma/hemangioma

Oropharyngeal dysplasia

Squamous cell carcinoma of the parotid gland

Oral melanocytic naevus/nevus

HER2 positive salivary gland cancer

**Osteonecrosis (SMQ)**

Dental necrosis

X-ray NOS face and mouth abnormal

Jaw lesion excision

Jaw pain/Pain in jaw

Dental abscess

Tooth infection

Oral surgery/NOS

Abscess jaw

Maxillofacial operation

Osteonecrosis of jaw

Jaw fistula

X-ray dental abnormal

Osteonecrosis

Bone pain/Pain bone

**Table S2. Comparison of the Individual Case Safety Reports (ICSRs) with at least one oropharyngeal adverse events between the period 2019-2021 and 2022-2024.**

| **Characteristics** | **Most reported level** | **Period 2019-2021** | **Period 2022-2024** |
| --- | --- | --- | --- |
| ICSRs | Number | 67,384 | 47,664 |
| ICSRs with O-AEs | Number | 2773 | 1740 |
| Sex | % Females | 71.0% | 65.7% |
| Age group | 18-64 years | 70.8% | 61.3% |
| Seriousness | Not serious | 68.4% | 66.7% |
| Outcome | Recovered/Resolved | 43.2% | 46.8% |
| Source | Physician/doctor | 53.8% | 74.4% |
| Suspected medicine | Drug vs Vaccine | 52.2% vs 47.8% | 90.5% vs 8.5% |
| O-AE | PT “Top twenty” | Throat tightness  Oral paresthesia  Oropharyngeal pain  Dysphagia  Dry mouth  Bone pain  Angioedema  Stomatitis  Aphthous ulcer  Oral herpes  Throat irritation  Osteonecrosis of the jaw  Hypoesthesia oral  Tongue edema  Swelling of tongue  Gingival bleeding  Oral disorder  Palatal edema  Oropharyngeal discomfort  Stevens-Johnson syndrome | Angioedema  Throat tightness  Stomatitis  Dry mouth  Dysphagia  Osteonecrosis of the jaw  Aphthous ulcer  Oropharyngeal pain  Oral Herpes  Oral candidiasis  Stevens-Johnson syndrome  Throat irritation  Salivary hypersecretion  Pharyngeal oedema  Pharyngitis  Oropharyngeal discomfort  Glossitis  Toxic epidermal necrolysis  Tongue discomfort  Gingivitis |
| ATC | First Level | “Antiinfectives for systemic use” (ATC J)  54.2% | “Antineoplastic and immunomodulating agents"  (ATC L) 46.8% |
